# Changes in Phytoplankton Community Composition and Phytoplankton Cell Size in Response to Nitrogen Availability Depend on Temperature

**DOI:** 10.3390/microorganisms10071322

**Published:** 2022-06-30

**Authors:** Veronika Dashkova, Dmitry V. Malashenkov, Assel Baishulakova, Thomas A. Davidson, Ivan A. Vorobjev, Erik Jeppesen, Natasha S. Barteneva

**Affiliations:** 1 School of Engineering and Digital Sciences, Nazarbayev University, Nur-Sultan 00010, Kazakhstan; 2 School of Sciences and Humanities, Nazarbayev University, Nur-Sultan 00010, Kazakhstan; dvmalashenkov@gmail.com (D.V.M.); assel.baishulakova@alumni.nu.edu.kz (A.B.); ivan.vorobyev@nu.edu.kz (I.A.V.); 3 National Laboratory Astana, Nur-Sultan 00010, Kazakhstan; 4 Department of Ecoscience, Aarhus University Center for Water Technology (WATEC), 8000 Aarhus, Denmark; thd@bios.au.dk (T.A.D.); ej@bios.au.dk (E.J.); 5 Sino-Danish Centre for Education and Research, Beijing 100049, China; 6 Limnology Laboratory, Department of Biological Sciences and Centre for Ecosystem Research and Implementation, Middle East Technical University, Ankara 06800, Turkey; 7 Institute of Marine Sciences, Middle East Technical University, Erdemli-Mersin 33731, Turkey; 8 The Environment & Resource Efficiency Cluster, Nazarbayev University, Nur-Sultan 00010, Kazakhstan

**Keywords:** phytoplankton, biodiversity, biovolume, cell size, eutrophication, mesocosm, temperature, nitrogen pollution, climate change, imaging flow cytometry

## Abstract

The climate-driven changes in temperature, in combination with high inputs of nutrients through anthropogenic activities, significantly affect phytoplankton communities in shallow lakes. This study aimed to assess the effect of nutrients on the community composition, size distribution, and diversity of phytoplankton at three contrasting temperature regimes in phosphorus (P)–enriched mesocosms and with different nitrogen (N) availability imitating eutrophic environments. We applied imaging flow cytometry (IFC) to evaluate complex phytoplankton communities changes, particularly size of planktonic cells, biomass, and phytoplankton composition. We found that N enrichment led to the shift in the dominance from the bloom-forming cyanobacteria to the mixed-type blooming by cyanobacteria and green algae. Moreover, the N enrichment stimulated phytoplankton size increase in the high-temperature regime and led to phytoplankton size decrease in lower temperatures. A combination of high temperature and N enrichment resulted in the lowest phytoplankton diversity. Together these findings demonstrate that the net effect of N and P pollution on phytoplankton communities depends on the temperature conditions. These implications are important for forecasting future climate change impacts on the world’s shallow lake ecosystems.

## 1. Introduction

Ongoing climate change strongly impacts freshwater aquatic ecosystems, altering trophic structure and dynamics [1,2,3,4], phenology, physiological and life-history traits of organisms, and intensifying the magnitude of eutrophication [2,4]. It involves shifts in many environmental factors (temperature, CO_2_ rise, nutrients loading) and significantly affects phytoplankton communities’ diversity, composition, and planktonic cell size in shallow lakes [5,6,7,8]. The prevalence of potentially toxic cyanobacteria in freshwater lakes is increasing, being favored under warmer and nutrient-rich conditions [9]. The harmful algal blooms (HABs) produced by cyanobacteria deteriorate water quality [10], and accelerate eutrophication by releasing more N and P into the environment [11]. 

However, the impact of climate change on phytoplankton communities’ composition and dynamics of freshwater ecosystems is not fully understood. Studies of phytoplankton communities’ composition and the taxonomic diversity of phytoplankton in response to warming and its consequences, e.g., increased nutrient concentration provide contrasting results [12,13,14,15,16]. In the short-term, warmer temperatures were shown to decrease phytoplankton diversity in temperate water bodies [12] and promote changes in the dominance and dynamics of phytoplankton species [13]. Long exposure to warming may result in the adaptation of phytoplankton species to warmer conditions [14], and an increase in functional phytoplankton diversity [15]. Warming-associated increased nutrient loading leads to decreased phytoplankton diversity [16]. It is reported that intensified N pollution in P-enriched eutrophic lakes may lead to changes in the phytoplankton composition, depending on the concentration of N input. Low-to-moderate N concentrations were seen to lead to the dominance of potentially toxic cyanobacteria (such as *Microcystis* spp., *Planktothrix* spp.) [17,18,19,20,21], and high concentrations of N may result in the prevalence of green algae [17,20]. 

Traditional microscopy is a method of choice in exploring changes in phytoplankton communities and phytoplankton cell size [22,23,24,25]; however, it is time-consuming with limited sampling capacity [26]. The phytoplankton communities’ structure is highly complex, and the use of other techniques such as spectral fluorometry and conventional flow cytometry is limited by a high degree of morphological heterogeneity and the microscopic size of the planktonic cells. Imaging flow cytometry (IFC) is a recent alternative and/or complementary approach to traditional microscopy. It allows for examining plankton communities, combining the large statistical power of flow cytometry and image-based analysis [27,28,29,30]. Large datasets of single-cell morphological and size parameters acquired via IFC enable a comprehensive evaluation of the response of plankton communities to environmental conditions [31,32,33,34]. It is a powerful technology for investigating microalgae [29] and enables the effective analysis of phytoplankton in a size range of ca. 10–300 µ [35]. To our knowledge, no research had explored the effect of N variation in different temperature scenarios on the dynamics of phytoplankton communities of shallow lake systems applying IFC.

This study aims to assess the temporal shifts in phytoplankton structure, biomass, size, and diversity in response to different N availability and temperature scenarios using eutrophic shallow lake mesocosms as a model. More specifically, we are interested in these questions: (1) Does N limitation leads to the dominance of phytoplankton taxa adapted to N-limited conditions, including N-fixing cyanobacteria? (2) Does N-limitation lead to decreased cell size and diversity due to the dominance of a few well-adapted species? (3) How does temperature variation impact phytoplankton community, particularly phytoplankton structure, biomass, cell/colony size, and diversity?

## 2. Materials and Methods

### 2.1. Experimental Setup

**Figure 1:**
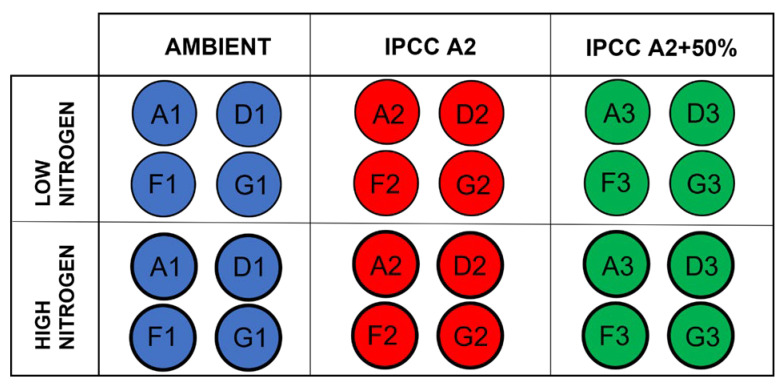
Schematic representation of the experimental design. Regular circles correspond to low nitrogen treatment (N0), and bold circles correspond to high nitrogen treatment (N+). Each temperature treatment has four replicate tanks: ambient temperature (A1, D1, F1, G1), IPCC A2 scenario (A2, D2, F2, G2), IPCC A2 + 50% scenario (A3, D3, F3, G3).

The study was conducted as part of the Lake Mesocosm Warming Experiment (LMWE) at the facility owned by Aarhus University, located at Lemming, Central Jutland, Denmark (56°14′ N, 9°31′ E). The LMWE has been running continuously since 2003 and it is the longest freshwater mesocosm experiment in the world. Overall, the facility includes 24 outdoor freshwater cylindrical steel tanks of 1.9 m diameter and 1.5 m depth with ventilated paddles installed at the bottom mixing the water to maintain uniform conditions. The water in mesocosms is sourced from groundwater with a water retention time of approximately 2.5 months [36]. The temperature regimes include unheated control with ambient water temperature (AMB) and two elevated temperature settings based on the IPCC climate scenarios for the period 2071–2100, IPCC A2 (ca. +3 °C) and IPCC A2 + 50% (ca. +4.5 °C), with four replications for each regime (AMB tanks: A1, D1, F1, G1; IPCC A2 tanks: A2, D2, F2, G2; IPCC A2 + 50% tanks: A3, D3, F3, G3) (Figure 1). 

The water temperature is maintained in the heated tanks using an automatic heating system with reference to the ambient air temperature on that daytime throughout the seasons. The current study was run in 12 mesocosms with high nutrient level which have been constantly supplied with 108.6 mg N per m^3^ per day and 2.7 mg P per m^3^ per day in the form Ca(NO_3_)_2_ and Na_2_HPO_4_, respectively. In total, each mesocosm was artificially supplied with 2152 mg N and 54 mg P per week in addition to the nutrient inputs from groundwater of approximately 2–5 mg P and 30–63 mg N per week. As a part of the experiment, N input was terminated in nutrient-rich tanks for one year to study the effect of N limitation on the lake community under euthrophic conditions. Resumption of N input the following year allowed us to study the response of the aquatic community to the N addition in terms of composition, biomass, size and diversity changes. In June 2018, the N supply in the high nutrient mesocosms was terminated for one year, maintaining the P input only. During this period, only insignificant quantities of N were supplied with the groundwater. In June 2019, the N additions were resumed along with continuing P addition. The initial average nutrient concentrations were TN 2.40 mg L^−1^ and TP 165 µg L^−1^ for the period 2017–2018 [37]. To study the response of phytoplankton community to N addition in eutrophic shallow lake systems, we compared the biological parameters of the high N period when N addition was resumed with the control low N period when N supply was terminated. The experimental design, sampling period and analysis procedures were identical in both treatments.

### 2.2. Sample Collection and Measurement of Water Parameters

Integrated water samples were collected using a 1 m long tube water sampler weekly or biweekly from the HN tanks from June to October in 2018 and in 2019. The samples collected for imaging flow cytometry and light microscopy were fixed with 1% glutaraldehyde for preservation before the analyses. Water temperature, turbidity, conductivity, oxygen, and pH were measured using YSI probes (Xylem Inc., College Station, TX, USA). Freshly collected unpreserved samples were analyzed for total nitrogen (TN), total phosphorus (TP), a sum of nitrites and nitrates (NO_2_ + NO_3_), ammonium (NH_4_), orthophosphates (PO_4_), and total Chl a as described in Søndergaard and co-authors [38], and total iron was measured spectrophotometrically (as described in Gibbs [39]). 

### 2.3. FlowCAM Analysis and Microscopy

A benchtop FlowCAM VS-4 imaging particle analyzer (Yokagawa Fluid Imaging Technologies, Scarborough, ME, USA) equipped with 532 nm excitation laser was used for the analyses. In total, 324 phytoplankton samples were collected and processed. The samples were analyzed in laser trigger mode using 10× and 20× objectives as described earlier [40]. The obtained particle images were classified into taxonomic phytoplankton groups based on image and size filters in Visual Spreadsheet software version 4.0 (Yokagawa Fluid Imaging Technologies, Scarborough, ME, USA) and manual inspection. The nanoplankton group based on 5–20 µm (diameter ABD) size filter was excluded from the analysis as it mostly contained cell debris, especially in dense blooming samples. Identifiable cell images under 20 µm were manually transferred to one of the morphological groups. Quantitative parameters of each group, including cell concentration and size parameters, were then obtained from the software, and used for biomass estimation and statistical analysis.

Taxonomic identification of phytoplankton cells and colonies was performed using a Leica DM2500 microscope (Leica Microsystems, Wetzlar, Germany) equipped with differential interference contrast (DIC). Phytoplankton species were identified using 63× and 100× objectives. 

### 2.4. Biovolume and Biomass Estimation

Size parameters such as width, length, diameter (ABD) were used for biovolume estimation of classified phytoplankton groups. Appropriate geometric shapes were assigned to the single cells and colonies of the present phytoplankton groups and biovolume was estimated using the corresponding formulae (according to Olenina [41], and Bergkemper and Weisse [42]. For unidentified species, FlowCAM biovolume estimations were based on ABD [43,44]. A full list of the shapes and formulae used is given in Table S1.

The biovolume estimates for most phytoplankton groups were obtained using size parameters retrieved from FlowCAM and standard microscopy biovolume formulae with some adaptations to two-dimensional image-based and colony-based size measurements (Table S1). The obtained biovolume estimates were converted to wet biomass units assuming that the plasma density equals 1 g cm^−3^. Mean values of single-cell biomass measurements for each phytoplankton group and date were obtained and converted into mg per L units. 

### 2.5. Statistical Data Analysis

The significance of water temperature variation between the two years was assessed using a non-parametric Kendall’s Tau b test available in SPSS software (IBM, Armonk, NY, USA). Multivariate analyses, including analysis of similarities ANOSIM, analysis of similarity percentages SIMPER, and PERMANOVA were performed using Primer-e software v.7 (PRIMER-E Ltd., Auckland, New Zealand). For ANOSIM and SIMPER, square root transformation was applied to raw data with average biomass values for each date from mid-June to September 2018 and 2019 for all phytoplankton groups, and the results were then converted to a resemblance matrix based on Bray–Curtis similarity distances. Two separate one-way (A) ANOSIM analyses based on Spearman correlation were performed to test the similarity of the phytoplankton community between the N0 and N+ treatments and between the temperature treatments. Bootstrap averages (150 bootstrap averages per group) were performed based on the resemblance matrix separately for N and temperature factor and displayed in mMDS plots. Square root transformed data was used to perform one-way SIMPER analysis based on Bray–Curtis similarity separately for N as a factor and temperature. Repeated measures PERMANOVA was performed on the data converted to a resemblance matrix based on binomial deviance with temperature and treatment as fixed factors and a time factor nested in the treatment. Permutation of residuals was performed using a reduced model with 9999 permutations, selecting type III (partial) sum of squares. To test the variation within the factor groups, PERMDISP, a distance-based test for homogeneity of multivariate dispersions, was also performed separately for each factor. The distances are calculated to centroids and *p*-values are obtained using permutations. 

Diversity indices were estimated as Pielou’s evenness (J′), Shannon–Wiener diversity index (H′), and Simpson index of diversity (1-D) applying Primer-e software. Diversity estimators were calculated for each tank and date from mid-June to September for the N0 and N+ treatments. Average values for each tank from mid-June to September during the N0 and N+ treatments were then calculated. Average values from the replicate tanks, corresponding to the AMB, IPCC A2, and IPCC A2 + 50% treatments, were then used to calculate the percentage change between the N0 and the N+ treatments. A Wilcoxon Signed Rank Test was used to test if the observed changes differed significantly from 0. 

Multivariate ordination analysis RDA was performed to infer relationships between phytoplankton data and environmental parameters using Canoco 5 software (Microcomputer Power, Ithaca, NY, USA). The response data had a gradient of 2.7 SD units for phytoplankton classes and 3.8 SD units for phytoplankton groups, suggesting that the optimum solution is to use a linear RDA method in the first case and a unimodal method CCA in the latter case. However, the two types of analyses may still be applied for both, and RDA was the preferred method. The analysis initially considered the data on 12 species and 11 environmental variables (TN, NO_2_ + NO_3,_ NH_4_, TP, PO_4_, TFe, Chl a, N:P, temperature, conductivity, turbidity). Before the analysis, the species data were log-transformed and centered by species. The significance of canonical axes was tested using 4999 time series permutations. Relationships between the phytoplankton groups and environmental factors were evaluated based on RDA bi-plots and explanatory response tables containing regression coefficients of phytoplankton group × environmental factor combination pairs.

Single-cell measurements of area-based (ABD) diameter retrieved from VisualSpreadsheet software for each tank from the period mid-June to September were used to estimate the relative frequency distribution as percentages using GraphPad Prism software (Dotmatics, Boston, MA, USA). The average of the single dates and standard deviation (SD) were calculated for each bin class for each tank for the N0 and N+ periods. Averages of the replicate tanks and SD were then calculated and used to plot the size distribution graphs. Descriptive statistics (mean, median, 25% percentile, 75% percentile) of the ABD measurements were obtained for the temperature treatments based on the average values of the replicate tanks and used to calculate the percentage change between the N0 and N+ treatments. The data were tested for normality using Anderson–Darling, D’Agostino–Pearson, Shapiro–Wilk, and Kolmogorov–Smirnov tests. One-sample *t*-test and Wilcoxon Signed Rank Test were used to test if the observed changes differed significantly from 0. 

## 3. Results

### 3.1. Environmental Changes

**Figure 2:**
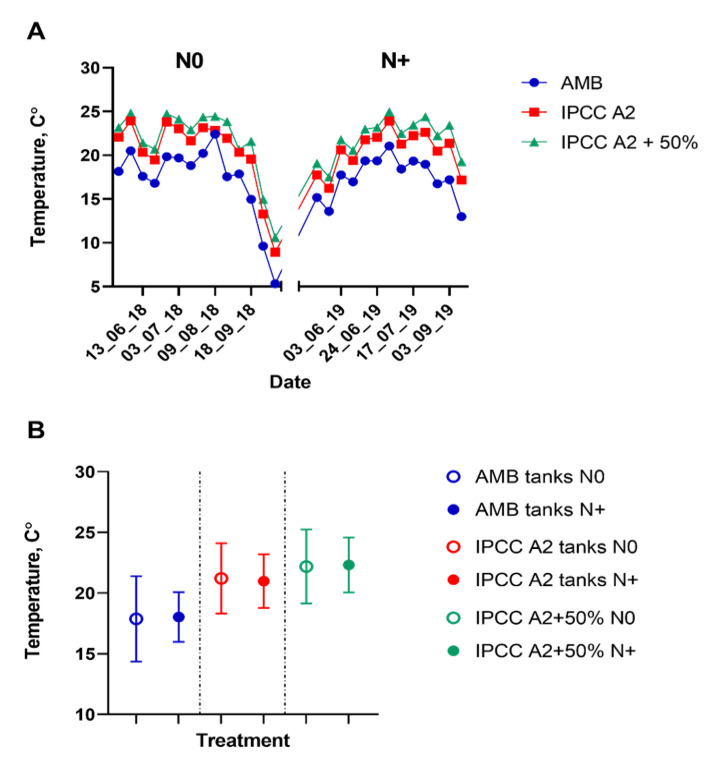
Water temperature measurements in the individual tanks. (**A**) Water temperature in the ambient temperature (AMB), IPCC A2, and IPCC A2 + 50% tanks from mid-June to September during the N0 and N+ treatments. (**B**) Mean water temperature for the mid-June–September period in AMB, IPCC A2 and IPCC A2 + 50% during the N0 and N+ treatments. Bars indicate SD of the mean.

The simulated temperature regimes (IPCC A2 and IPCC A2 + 50% scenarios) differed, as expected, from the ambient temperature during the study period (Figure 2). 

Mean water temperature did not differ significantly between the N0 and N+ sampling seasons (Figure 2; Table S2), implying that the observed differences in phytoplankton composition between the two treatment regimes can be attributed to the experimental conditions and not natural year-to-year variations in temperature. 

**Figure 3:**
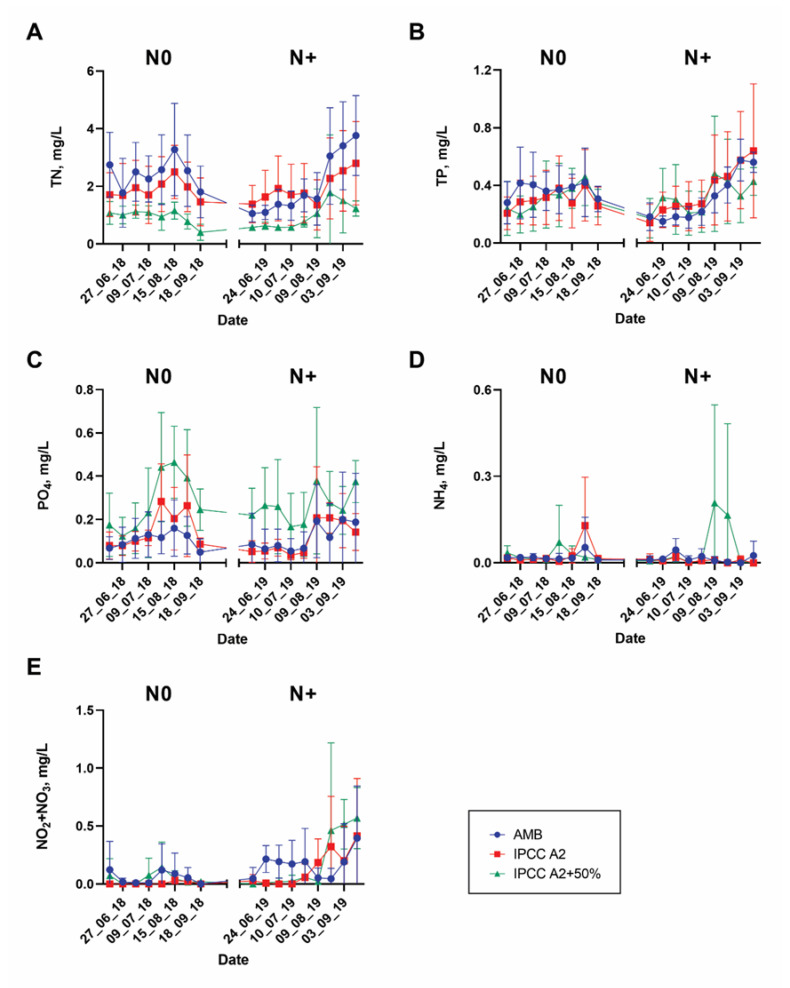
Variations in nutrients from mid-June to September during the N0 and N+ periods: (**A**) TN; (**B**) TP; (**C**) PO_4_; (**D**) NH_4_; (**E**) NO_2_ + NO_3_. Bars—SD of the mean.

Variations of the main environmental variables, including total nitrogen (TN), inorganic forms of nitrogen (NO_2_ + NO_3_ and NH_4_), total phosphorus (TP), and phosphates (PO_4_), in the N0 and N+ treatments are displayed in Figure 3 and Figure S1. 

Overall, large variability in nutrient concentrations among the individual replicate tanks was observed (Figure 3). Thus, TN and PO_4_ concentrations differed between the temperature treatments. TN was relatively higher in AMB than in IPCC A2 + 50%, whereas PO_4_ was the highest in IPCC A2 + 50% (Figure 3A,C). However, no clear effect of N treatment on TN was observed; TN varied between 0–4.3 mg/L and 0.1–5.4 mg/L during the N0 and N+ treatments, respectively. By contrast, NO_2_ + NO_3_ tended to increase in all temperature treatments after N addition resuming and was significantly higher in AMB and IPCC A2 than in IPCC A2 + 50%. NH_4_, TP, and PO_4_ concentrations did not respond significantly to the N treatment. 

### 3.2. Total Phytoplankton Biomass and Composition

**Figure 4:**
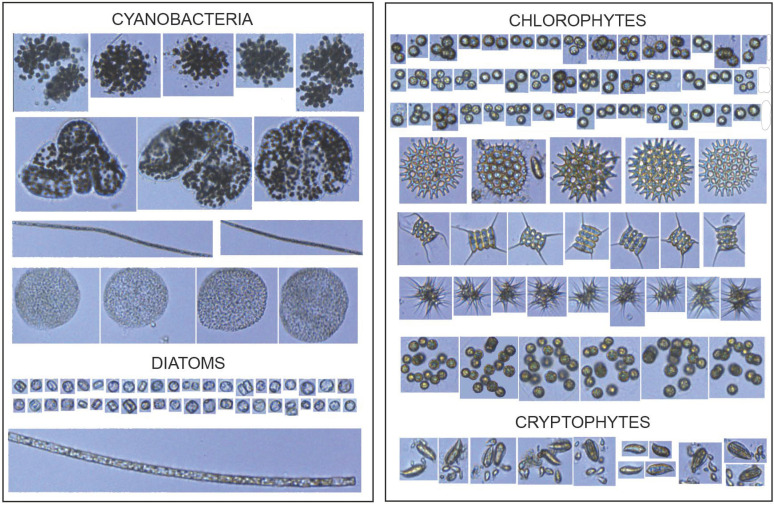
FlowCAM image library showing the different phytoplankton groups (10× objective).

The phytoplankton community was composed of taxonomic groups belonging to Cyanophyta, Chlorophyta, Cryptophyta, Miozoa, Bacillariophyta, and Euglenozoa phyla (Table S3; Figure 4). 

Sixteen major morphologically distinct groups were classified using a FlowCAM imaging flow cytometer (Table S3). 

**Figure 5:**
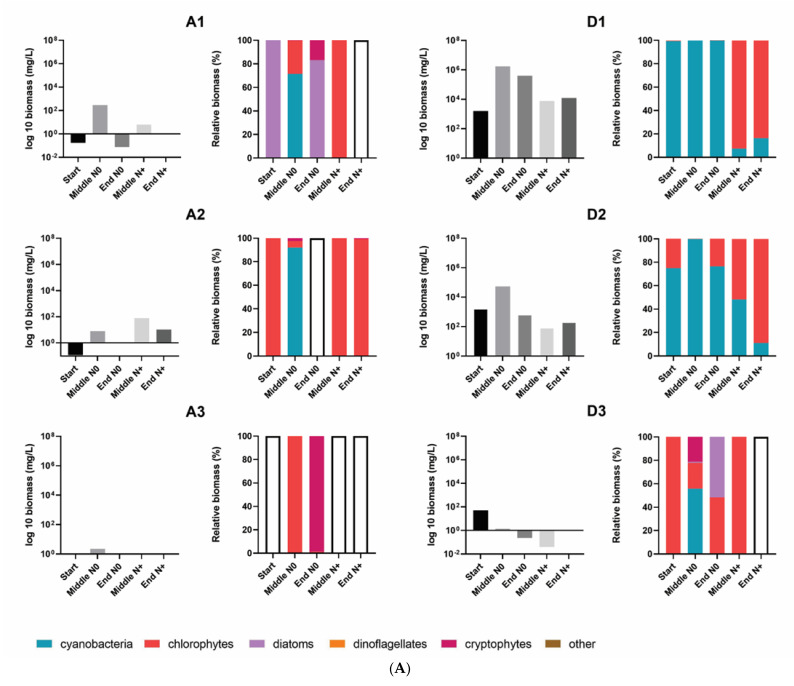

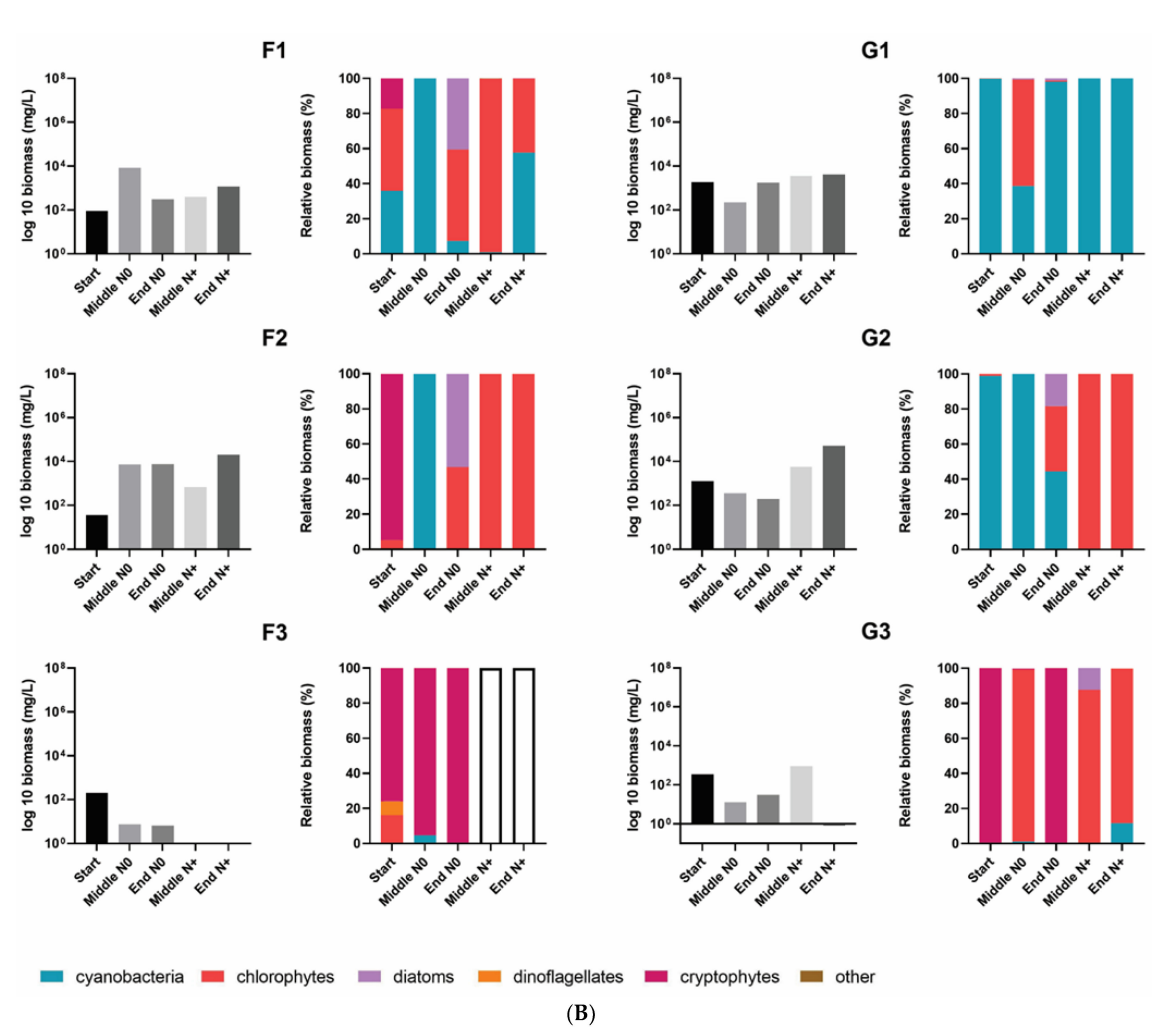
Total phytoplankton biomass and the relative contribution of the different phytoplankton groups to total biomass in the individual tanks at the start, in the middle and at the end of the N0 treatment and in the middle and at the end of the N+ treatment. (**A**) A1–A3, and D1–D3 individual tanks; (**B**) F1–F3, and G1–G3 individual tanks. Empty bars indicate samples with no cell biomass.

Total phytoplankton biomass varied significantly among the individual tanks. Generally, the lowest total phytoplankton biomass was found in the A tanks (A1, A2, A3) and in the tanks with the highest temperature (IPCC A2 + 50%). A1–A3 tanks had an extremely low cell biomass throughout the experiment and occasional maximum peak values were associated with the development of cyanobacteria (A1 = 290 mg/L) and chlorophytes (A2 = 79 mg/L) (Figure 5A,B). 

The community biomass of IPCC A2 + 50% was mainly composed of cryptophytes and chlorophytes. Cryptophytes dominated the total biomass in the N0 treatment and had several blooms; however, when the N supply was resumed a shift in dominance to chlorophytes occurred (Figure 5). 

Except for A1–A2 tanks, AMB and IPCC A2 temperature tanks had relatively higher phytoplankton biomasses, reaching 17 × 10^5^ mg/L of total biomass during blooming events in some of the tanks (Figure 5). Although there was no uniform response of total biomass to the N treatment in AMB and IPCC A2, common trends in phytoplankton composition occurred. During the N0 treatment cyanobacteria dominated the summer community in most of the AMB and IPCC A2 tanks, whereas the following summer when N supply was resumed the contribution of chlorophytes to the total biomass increased significantly, except for the G1 tank (Figure 5). Cyanobacteria biomass mostly consisted of filamentous and potential N-fixing *Cuspidothrix* spp., *Pseudanabaena* spp., and non-N-fixing colony-forming *Microcystis* spp. (Figure S2). As expected, N-fixing filamentous cyanobacteria, including *Cuspidothrix* sp., had several major outbreaks in summer and autumn during the N0 treatment in D1 and D2. While the biomass of filamentous cyanobacteria decreased significantly in the N+ period, *Microcystis* spp. did not show a uniform response to resumed N addition in terms of biomass. Despite the fact *Microcystis* spp. still dominated total community biomass in several tanks (D2, G1, G2) after the N supply was resumed, the magnitude of the biomass outbreaks decreased. In the N+ treatment, the proportion of chlorophytes, mainly *Micractinium* spp. and *Pediastrum* spp. (Figure S2), of total community biomass increased. Blooming events of *Micractinium* spp. developed towards the end of summer in most of the tanks. 

### 3.3. Changes in Phytoplankton Community Structure

**Figure 6:**
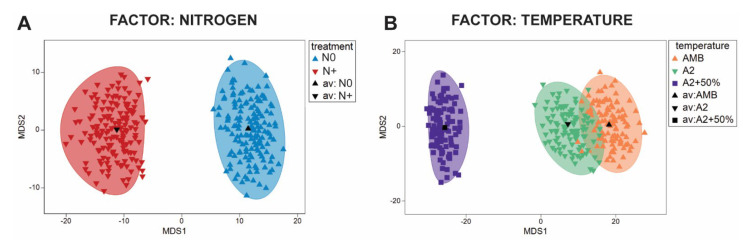
Bootstrap regions for phytoplankton community averages plotted in an mMDS plot with 150 bootstraps per group and 95% coverage. (**A**) Nitrogen treatment factor; (**B**) Temperature treatment factor.

According to the ANOSIM analysis of similarities, there were significant differences in the entire phytoplankton community between the N0 and N+ treatments (R = 0.069, *p* < 0.001). The phytoplankton community of IPCC A2 + 50% differed significantly different from that of AMB (R = 0.412, *p* < 0.001) and A2 (R = 0.293, *p* < 0.001), while there was no significant difference between AMB and IPCC A2 (R = −0.002, *p* = 0.45). The observed dissimilarities are graphically illustrated in Figure 6 (below) using estimated bootstrap averages based on Bray–Curtis similarity matrix in mMDS space.

**Figure 7:**
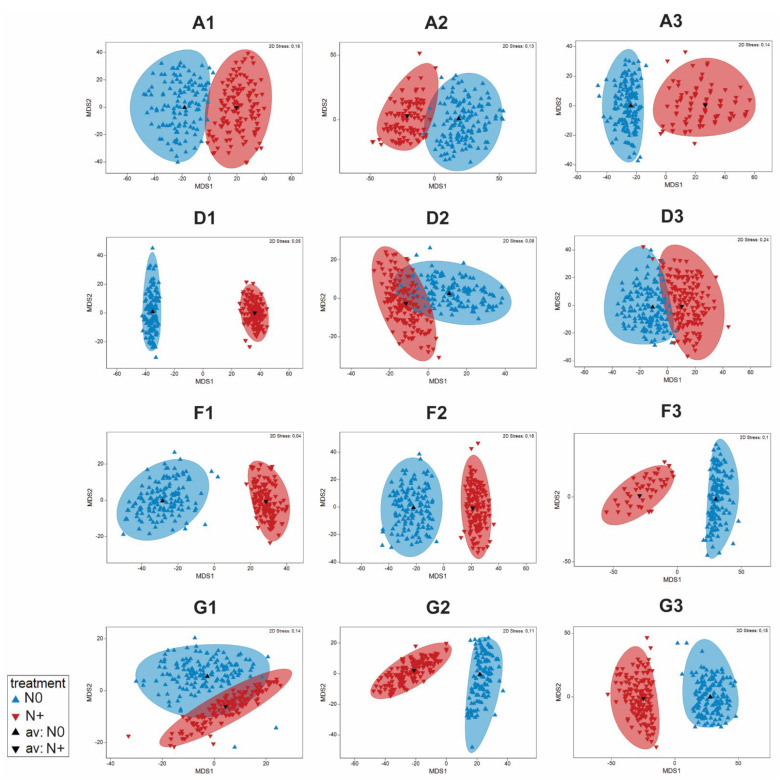
Bootstrap regions for phytoplankton community averages for tanks A1–A3, D1–D3, F1–F3, and G1–G3 plotted in an mMDS plot with 150 bootstraps per group and 95% coverage.

ANOSIM analysis of the individual tanks showed significant differences between the N0 and N+ treatments for majority of tanks (A1: R = 0.068, *p* < 0.17; A2: R = 0.109, *p* < 0.11; A3: 0.168, *p* < 0.03; D1: R = 0.836, *p* < 0.001; D2: R = −0.003, *p* < 0.43; D3: R = −0.086, *p* < 0.92; F1: R = 0.58, *p* < 0.002; F2: R = 0.272, *p* < 0.004; F3: R = 0.177, *p* < 0.03; G1: R = −0.005, *p* < 0.4; G2: R = 0.248, *p* < 0.012; G3: R = 0.285, *p* < 0.003). No significant differences were found between the treatments attributed to A tanks and all IPCC + 50% tanks, which can be explained by low cell biomass in those tanks. Additionally, there is some variability among the replicate tanks differing in the extent of the community dissimilarity between the treatments. Dissimilarities based on bootstrap regions for the phytoplankton communities from each tank are displayed in Figure 7.

PERMANOVA analysis confirmed the significant effect of singular factors, such as N variation (PERMANOVA, pseudo-F = 6.02, *p* < 0.003) and temperature (PERMANOVA pseudo-F = 4.2, *p* < 0.05), on the phytoplankton taxonomic structure, but also revealed a significant interaction effect of N and temperature variation (PERMANOVA, pseudo-F = 4, *p* < 0.05). Moreover, significant variation in phytoplankton structure was found among the N (PERMDISP, F = 7.6, *p* < 0.0001) and the temperature (PERMDISP, F = 6.7, *p* < 0.0001) treatments.

Analysis of similarity percentages, SIMPER, was performed to identify the phytoplankton groups creating dissimilarity among the treatments. Overall, biomass changes in cyanobacteria, chlorophytes, and cryptophytes contributed mostly to the differences observed between the N treatments and the temperature regimes (Table S4). However, the exact contribution percentage to the dissimilarity varied among the tanks. Thus, cyanobacteria biomass decreased during the N+ treatment compared to N0 treatment, whereas the biomass of chlorophytes increased after N addition was resumed. In IPCC A2 + 50%, cryptophytes were added to the groups accounting for the dissimilarity between treatments; they were almost exclusively found in the tanks with the highest temperature. The differences in community composition between the temperature treatments were also caused by the biomass variations of cyanobacteria and chlorophytes (Table S4).

### 3.4. Changes in Phytoplankton Size Distribution

**Figure 8:**
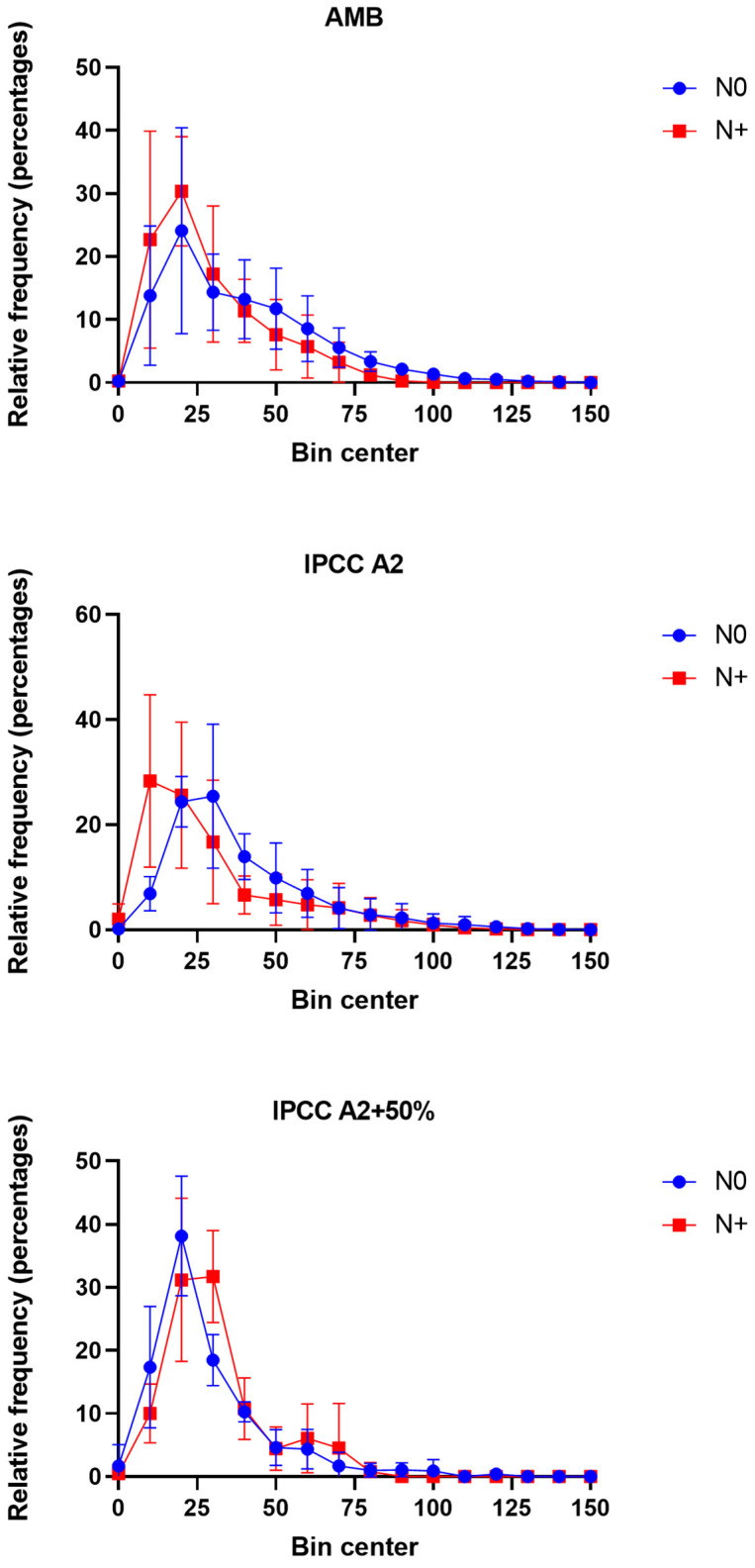
Phytoplankton (cell and colony) size distributions in AMB, IPCC A2, and IPCC A2 + 50% in the N0 and N+ treatments based on the average distributions of area-based diameter (ABD) in the individual tanks for the period mid-June–September. Each bin contains the number of size values in certain range of values. The bin width equals 10. Bars—SD of the mean.

Single-cell measurements of area-based diameter (ABD) were used to obtain size frequency distributions for the AMB, IPCC A2, and IPCC A2 + 50% temperature treatments to evaluate changes in phytoplankton community size in response to N addition (Figure 8). 

The size medians in AMB and IPCC A2 were similar and showed a decrease from 37 to 26 µm in AMB and from 37 to 28 µm in IPCC A2 in response to the N addition (Figure 8). In contrast, the size median in IPCC A2 + 50% tanks from 22 to 31 µm during the N+ treatment (Figure 8). The same pattern was observed for other descriptive statistics indicators, including ABD diameter mean, and 25% and 75% percentiles. 

### 3.5. Changes in Phytoplankton Diversity

**Figure 9:**
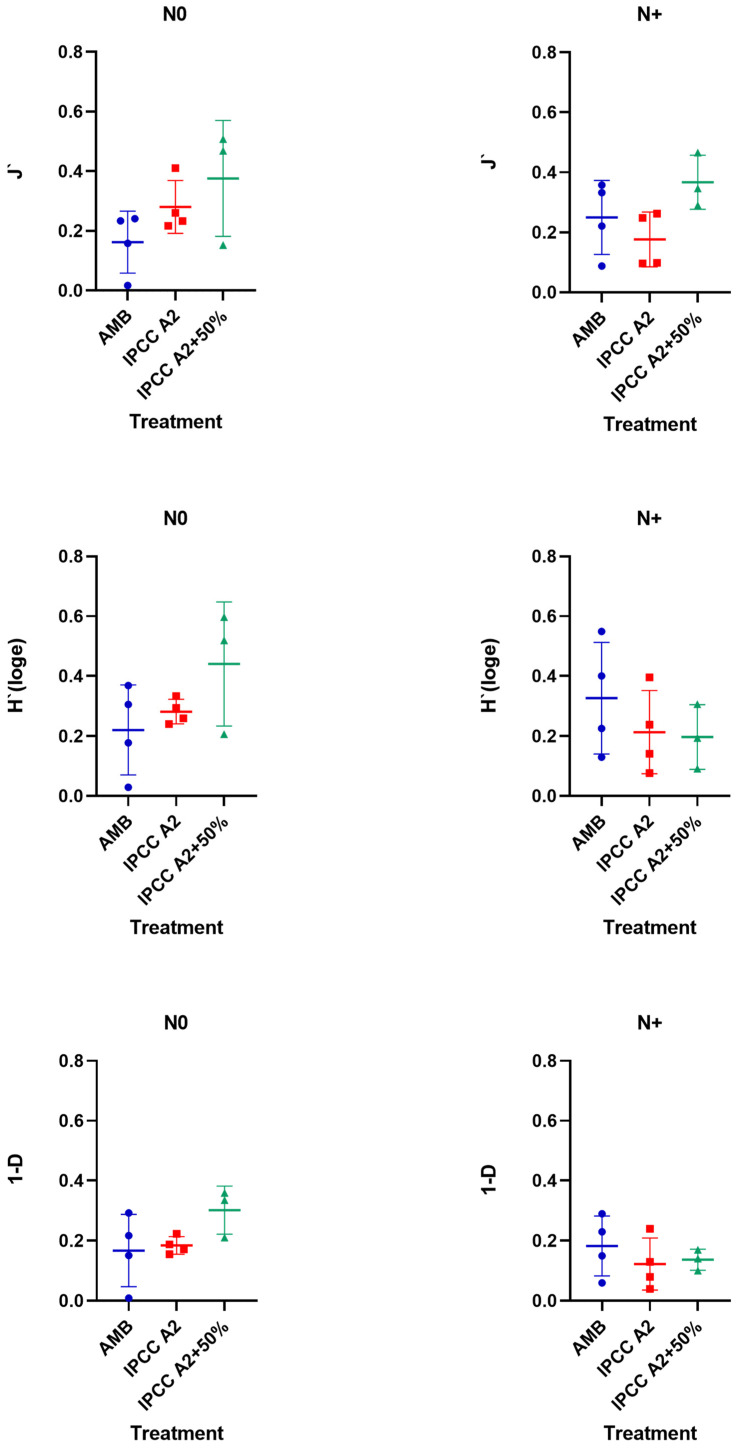
Phytoplankton diversity estimated using Pielou’s evenness (J′), Shannon–Wiener diversity index (H′), and Simpson diversity index (1-Lambda (D)) in AMB, A2, and A2 + 50% in the N0 and +N treatments.

Changes in phytoplankton diversity among the treatments were assessed as a total of Pielou’s evenness (J′), Shannon–Wiener diversity index (H′), and Simpson diversity index (1-Lambda (D)) (Figure 9).

In the N0 treatment, the highest diversity was observed in IPCC A2 + 50% (Figure 9) compared to AMB and IPCC A2. Whereas the J′ index value associated with community evenness did not change in the IPCC A2 + 50% treatment, the H′ and 1-D indices decreased almost twice as much when N addition was resumed (Figure 9). Similarly, phytoplankton diversity tended to decline in IPCC A2, while AMB demonstrated a slight diversity increase in the N+ treatment (Figure 9). The highest diversity based on H′ and 1-D indices was observed in AMB in the N+ treatment (Figure 9).

### 3.6. RDA of Phytoplankton Community and Environmental Factors

**Figure 10:**
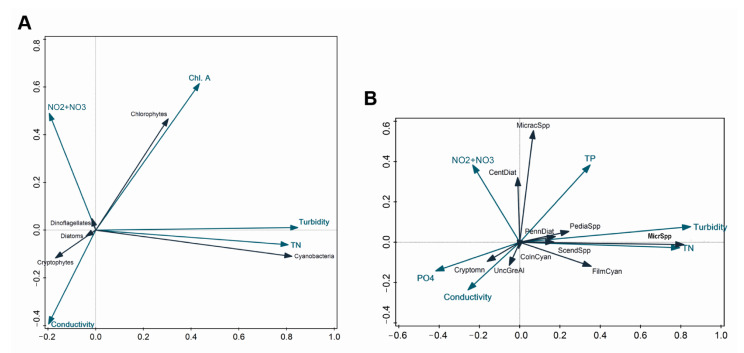
RDA plots for phytoplankton biomass for the whole study period. (**A**) phytoplankton presented as classes; (**B**) phytoplankton presented as groups. Blue arrows—environmental parameters; black arrows—phytoplankton taxa: MicracSpp—*Micractinium* spp., PennDiat—pennate diatoms, PediaSpp—*Pediastrum* spp., MicrSpp—*Microcystis* spp., ScendSpp—*Scenedesmus* spp., FilmCyan—filamentous cyanobacteria, ColnCyan—colonial cyanobacteria, UncGreAl—unicellular green algae, Cryptomn—cryptomonads. Only significant environmental factors (*p* < 0.05) are displayed. Graphical relationships between different parameters reflect the contribution percentage of each parameter accounting for simple effects, which vary from the percentage contribution based on the conditional effects, used for results interpretations.

We performed an RDA analysis to evaluate the relative contribution of different environmental factors to the phytoplankton community variation. Overall, the available environmental variables explained 48.3% and 40.0% of the phytoplankton biomass variation in the RDA performed for phytoplankton classes and groups, respectively (Figure 10). 

In both cases, turbidity contributed most to the total community variation (28.5%, *p* < 0.004 and 19.6%, *p* < 0.004, respectively), followed by Chl a, conductivity, TN concentration, and NO_2_ + NO_3_ concentration, each of which contributed less than 7.4% (Figure 10A,B). Additionally, TP (1.2%, *p* < 0.04) and PO_4_ (1.4%, *p* < 0.02) concentrations contributed significantly to the variation among the phytoplankton groups (Figure 10B). 

Several significant correlations appeared between the biotic data and environmental variables. Turbidity correlated positively with the biomass of both *Microcystis* spp. and, to a lesser extent, filamentous cyanobacteria. Similarly, the biomass of cyanobacteria, including *Microcystis* spp. and filamentous cyanobacteria, was associated with high TN (Figure 10A, B) (*R*^2^ ≥ 0.9, *p* < 0.01). There was a negative relationship between NO_2_ + NO_3_ and the biomass of *Microcystis* spp., whereas the opposite pattern was observed for *Micractinium* spp. However, the strength of these relationships was weak (*R*^2^ ≤ 0.18, *p* < 0.01). Both *Microcystis* spp. and *Micractinium* spp. correlated positively with TP (*R*^2^ = 0.39 and 0.18, respectively, *p* < 0.01), while there was a strong negative relationship between *Microcystis* spp. and PO_4_ (*R* = −0.66, *R*^2^ = 0.43, *p* < 0.01) and, to a lesser extent, between *Micractinium* spp., filamentous cyanobacteria, and PO4 (*R*^2^ ≤ 0.07, *p* < 0.01). 

## 4. Discussion

Temperature-related shifts in algal communities have already been demonstrated in a number of natural phytoplankton community studies [23,45,46]. Classically, the increasing dominance of large-size phytoplankton species is highly dependent on the higher input of new nutrients. In the last decade, IFC has been used to assess the composition, abundance, size, and biovolume of phytoplankton and zooplankton in mesocosm experiments [47,48,49,50,51,52]. The IFC-based analysis allowed us to record frequently phytoplankton population dynamics based on biomass and cell size of thousands of cells in 16 phytoplankton groups in response to N and temperature variation. However, to our knowledge, no studies have explored the dynamics of phytoplankton communities of shallow lake systems in response to the combined treatment of N and temperature variation using IFC technology. We believe that this work may contribute to understanding climate change and nutrient pollution effects on shallow freshwater ecosystems. 

### 4.1. Phytoplankton Cell Size

Phytoplankton cell size is an essential physiological parameter influencing food web structure, the efficiency of energy transfer, and the flux of carbon in aquatic ecosystems (ref. [53]). It was reported before that phytoplankton cell size decreases with warming ([54,55]), whereas increased nutrient availability is predicted to promote bigger cell sizes [56] and greater size diversity.

In the LMWE experiment, we observed a decrease in phytoplankton size in AMB and IPCC A2 after resuming the N addition. The reduced cell size may reflect the shift in dominance from *Microcystis* colonies to *Micractinium* single cells and colonies, with *Micractinium* being smaller in size. Generally, the average cell size in the highest temperature tanks was smaller than in AMB and IPCC A2, and there was a significant difference between AMB and IPCC A2 + 50% in the N0 period. In contrast, the average cell/colony size increased in the tanks with the highest temperature regimes when the N supply was resumed. Assuming that a relatively smaller size is the survival strategy adopted by phytoplankton populations under high-temperature stress and nutrient deficiency [57], the availability of N favored the greater size diversity. However, as in the other temperature treatments, the shift in size distribution may be attributed to the replacement of cryptomonads by relatively larger colonies of *Micractinium* spp. rather than size changes within the phytoplankton classes.

### 4.2. Phytoplankton Community Composition and Biomass Shifting

We applied IFC to assess phytoplankton composition and diversity, biomass, and size of planktonic cells. Recorded images were classified into 16 distinct groups based on morphological features (Table S3) with accuracy comparable to the measurements obtained by traditional microscopy [27,32,58]. However, due to the estimation algorithm using the area based on the two-dimensional images, instead of true three-dimensional shapes, biovolumes obtained by IFC tend to be misestimated [58]. The alternative is to use linear dimensions derived from taxonomic information on a particular cell to apply a shape-specific biovolume estimation in microscopy [42,58,59]. Yet, in the case of complicated shapes such as those of *Desmodesmus* spp. and/or unidentified species, FlowCAM-derived ABD volumes with the appropriate thresholding of the dark and light pixels of the images were applied.

Different temperature regimes led to shifts in phytoplankton community composition and biomass. The lowest biomass was found at ca. 4.5 °C above the ambient temperature and all A tanks. The latter observation can be explained by the presence of macrophytes in those tanks compared with the other introduced by chance. Phytoplankton community composition at ca. 4.5 °C above the ambient temperature was different from the community at ambient temperature and ca. +3 °C temperature. Phytoplankton community composition in ambient and ca. +3 °C temperature did not differ significantly and responded similarly to the variation in N. Mesocosms at both temperature regimes were dominated by cyanobacteria and green algae, whereas mesocosms with the highest temperature regime were mostly dominated by cryptomonads and green algae. The phytoplankton composition and dynamics in the high-nutrient tanks resembles those in the natural eutrophic lakes [60,61,62]. Bloom-forming *Microcystis* spp. and *Micractinium* spp. were the most abundant representatives of Cyanobacteria and Chlorophyta, respectively. The resuming of the N supply in P-enriched mesocosms led to a significant increase in the proportion of green algae, including the genus *Micractinium* spp., and a decrease in the proportion of cyanobacteria in AMB and IPCC A2 temperature regimes. 

A shift towards dominance by green algae of the phytoplankton community with increasing N has been reported in mesocosm studies simulating shallow lakes [63] and natural field samples in shallow lakes at high N loading [17,64,65,66]. Despite the well-recognized harm of cyanobacteria blooms to the aquatic ecosystem, the blooming of other phytoplankton groups may also have serious implications for the ecosystem’s biodiversity and functioning [67,68]. In their recent paper, Amorim and do Nascimento Moura [68] showed that both one-type and mixed (Cyanobacteria and other algal taxa) blooms could impact water quality, biodiversity, trophic dynamics, and ecosystem functioning. Our results demonstrate that variation in the amount of N in P-enriched systems may shift the dominance between the bloom-forming cyanobacteria and cyanobacteria/green algae. Because of the expected intensification of algal blooms in the future years due to climate change [69], management efforts should be concentrated on the mixed-type blooms as well.

We found a positive correlation between cyanobacteria and TN amount, despite the observed prevalence of cyanobacteria in N-limited conditions, likely reflecting overall low inorganic N concentration, with most of the N being stored in the phytoplankton. Moreover, the relationships between TP, PO_4_, and *Microcystis* spp. showed opposite patterns. *Microcystis* spp. correlated positively with TP and negatively with PO_4_. Different relationships between TP, PO_4_, and cyanobacteria have been shown previously (e.g., [70]), resulting from the fact that TP includes internal stores of phosphorus in phytoplankton, while PO_4_ represents soluble reactive phosphorus, which is affected by many processes including sediment–water interactions [71]. 

### 4.3. Phytoplankton Diversity

The contrasting patterns observed for the different temperature regimes imply an interaction effect of temperature and N availability on phytoplankton diversity. Phytoplankton diversity tended (though not significantly so) to increase in AMB and decrease in IPCC A2 and IPCC A2 + 50% after resuming the N supply. The combination of high temperature and high N led to the lowest diversity compared with the N0 conditions, where the highest diversity was observed in the IPCC A2 + 50% tanks (Figure 9). Studies of the effect of temperature on diversity have yielded contrasting results. In a mesocosm experiment, Yvon-Durocher and colleagues [72] observed a 67% increase in phytoplankton species richness and evenness in response to 4 °C warming. A field study also found increases in phytoplankton diversity with warming; however, in this case, it was possibly attributable to increased nutrient concentrations [73]. Other studies revealed a reduction in phytoplankton diversity with warming [74,75]. However, it was also shown that the effect of warming on biodiversity might vary with the nutrient levels [23,73,76], as in our study. Based on results, it is evident that temperature increase accompanied by increased nutrient pollution will negatively impact phytoplankton diversity.

## 5. Conclusions

In conclusion, we were able to capture the response of phytoplankton communities to N availability under contrasting temperature conditions using IFC. Our findings showed that N variation in P-enriched environments profoundly affects the composition, biomass, size distribution, and diversity of phytoplankton communities. Moreover, the net impact of N loading on the phytoplankton community depended on the temperature conditions. Based on the temperature regime, the phytoplankton community had a differential response to N variation in species composition, cell size, and diversity, changing the dominance between the bloom-forming cyanobacteria and cyanobacteria/green algae. The average cell size in the highest temperature tanks was smaller but increased in the tanks with the highest temperature regimes when the N supply was resumed. Therefore, although it adds to the complexity, it is important to consider the interaction of nutrient level with temperature when assessing the impact of nutrient loading on freshwater ecosystems in the context of climate change. Our results demonstrate that the IFC-based approach can be a valuable tool in capturing the dynamics of structural and morphological changes in phytoplankton communities.

## Data Availability

The data that support the findings of this study are available from the corresponding author, upon reasonable request.

## Supplementary Materials

The following supporting information can be downloaded at: https://www.mdpi.com/article/10.3390/microorganisms10071322/s1, Table S1: Shapes and formulae used for biovolume estimation; Table S2: Average temperature data for temperature treatment tanks for N0 and N+ periods during June–October and correlation results between the two datasets obtained using Kendall’s Tau test; Table S3: Phytoplankton composition in the high-nutrient tanks captured by FlowCAM imaging flow cytometer and microscopy; Table S4: SIMPER analysis results for different temperature tanks; Figure S1: Concentrations of the main nutrients (TN, NH4, NO2+NO3, TP, PO4) in the tanks exposed to different temperature treatments (AMB, IPCC A2, IPCC A2+50%); Figure S2: Relative contribution of the different phytoplankton groups to the total biomass in the individual tanks during the N0 and +N treatments.
