# Supplementary material for: Changes in Phytoplankton Community Composition and Phytoplankton Cell Size in Response to Nitrogen Availability Depend on Temperature
Source: Microorganisms. 2022 Jun 30;10(7):1322. doi: 10.3390/microorganisms10071322 (PMC9324377; doi:10.3390/microorganisms10071322)
Supplement: Supplementary file 1 [file microorganisms-10-01322-s001.zip › microorganisms-1737733-supplementary.pdf]

Supplementary Materials

Changes in Phytoplankton Community Composition and Phytoplankton Cell Size in Response to Nitrogen Availability Depend on Temperature

Veronika Dashkova [1,2,*], Dmitry V. Malashenkov [2,3,†], Assel Baishulakova [2], Thomas A. Davidson [4], Ivan A. Vorobjev [2], Erik Jeppesen [4,5,6,7] and Natasha S. Barteneva [2,8,*]

1 School of Engineering and Digital Sciences, Nazarbayev University, Nur-Sultan 00010, Kazakhstan
2 School of Sciences and Humanities, Nazarbayev University, Nur-Sultan 00010, Kazakhstan; dvmalashenkov@gmail.com (D.V.M.); assel.baishulakova@alumni.nu.edu.kz (A.B.); ivan.vorobyev@nu.edu.kz (I.A.V.)
3 National Laboratory Astana, Nur-Sultan 00010, Kazakhstan
4 Department of Ecoscience, Aarhus University Center for Water Technology (WATEC), 8000 Aarhus, Denmark; thd@bios.au.dk (T.A.D.); ej@bios.au.dk (E.J.)
5 Sino-Danish Centre for Education and Research, Beijing 100049, China
6 Limnology Laboratory, Department of Biological Sciences and Centre for Ecosystem Research and Implementation, Middle East Technical University, Ankara 06800, Turkey
7 Institute of Marine Sciences, Middle East Technical University,33731, Erdemli-Mersin, Turkey
8 The Environment&Resource Efficiency Cluster, Nazarbayev University, Nur-Sultan 00010, Kazakhstan
* Correspondence: veronika.dashkova@nu.edu.kz (V.D.); natalie.barteneva@nu.edu.kz (N.S.B.)
† Current address: Department of Hydrobiology, Faculty of Biology, Moscow State University, 119991 Moscow, Russia.

Table S1. Shapes and formulae used for biovolume estimation.

**Phytoplankton group**	**Shape applied**	**Single cell or colony**	**Formula used**	**Adapted formula**
Microcystis novacekii **(Komárek) Compère 1974**	Cylinder	Colony	3.1416*(((D (ABD)/2)^2)/1.5)*single cell D	3.1416*(((D (ABD)/2)^2)/1.5)*7
M. aeruginosa, M. wesenbergii, M.flos-aquae, M.smithii	Cylinder	Colony	3.1416*(((D (ABD)/2)^2)/1.5)*single cell D	3.1416*(((D (ABD)/2)^2)/1.5)*4
Pediastrum **spp.**	Cylinder	Colony	3.1416/4*D^2*H	3.1416/4*W^2*L
Desmodesmus **spp. (*Scenedesmus* spp.)**		Colony	Vol (ABD)	Vol (ABD)
Micractinium **spp.**	Sphere	Colony	3.1416/6*D^3	3.1416/6*W^3

**Dinoflagellates**	Rotational ellipsoid	Cell	3.1416/6*D^2*H	3.1416/6*W^2*L
**Filamentous cyanobacteria**	Cylinder	Colony	3.1416/4*D^2*H	3.1416/4*W^2*L
**Cryptomonads**	Flattened ellipsoid	Cell/colony	3.1416/6*D1*D2*H	3.1416/6*W*(W/2)*L
**Pennate diatoms (*Nitzschia* sp.)**	Diamond box	Cell	W*W*(L/2)	W*W*(L/2)
**Unicellular green algae *Monoraphidium* sp.**	Double cone	Colony	3.1416/12*D^2*H	(3.1416/12*2,5^2*25)*4
**Centric Diatom**	Cylinder	Cell/Colony	3.1416/4*D^2*H	3.1416/4*W^2*L
***Aphanocapsa* spp. (colonial cyanobacteria)**	Cylinder	Colony	3.1416*(((D (ABD)/2)^2)/1.5)*single cell D	3.1416*(((D (ABD)/2)^2)/1.5)*2
***Micractinium* spp.**	Sphere	Colony and cell	3.1416/6*D^3	3.1416/6*W^3
Sphaerocystis* sp.	Cylinder	Colony	3.1416*(((D (ABD)/2)^2)/1.5)*single cell D	3.1416*(((D (ABD)/2)^2)/2)*14
**Colonial cyanobacteria unidentified**	Volume ABD	Colony or multiple cells	Vol (ABD)	Vol (ABD)
***Closterium* sp.**	Double cone	Cell	3.1416/12*D^2*H	3.1416/12*W^2*L
***Cosmarium* sp.**	Ovoid	Cell	(4/3)*3.1416*(W/2)^2*(L/2)	(4/3)*3.1416*(W/2)^2*(L/2)
***Monoraphidium* sp. single cell**	Double cone	Cell	3.1416/12*D^2*H	3.1416/12*W^2*H

D – Diameter; H – Height; L – Length; W – Width; ABD – area-based diameter.

Table S2. Average temperature data for temperature treatment tanks for N0 and N+ periods during June – October and correlation results between the two datasets obtained using Kendall`s Tau test.

N0 period			N+ period			Treatment	Correlation Coefficient	Sig. (2-tailed)	N
AMB N0	A2N0	A2+50 N0	AMB N+	A2N+	A2+50 N+	AMBN0 vs. AMBN+	0.455	0.052	11
17.6	20.3375	21.435	17.75	20.6075	21.7625	A2N0 vs. A2N+	0.345	0.139	11
16.805	19.4725	20.7075	16.96	19.4025	20.545	A2+50N0 vs. A2+50N+	0.382	0.102	11
19.835	23.815	24.74	19.3725	21.7625	22.985				
19.7025	23.0375	24.13	19.3625	22.0475	23.1725				
18.82	21.655	22.905	21.055	23.905	24.97				
20.21	23.155	24.3575	18.4225	21.2625	22.4875				
22.415	22.8375	24.455	19.3375	22.24	23.47				
17.5575	21.9425	23.8425	18.9725	22.62	24.4075				
17.855	20.3475	20.665	16.7375	20.47	22.245				
14.96	19.57667	21.6	17.2	21.365	23.42				
9.6125	13.29	14.9725	12.9975	17.1825	19.27				

Table S3. Phytoplankton composition in the high-nutrient tanks captured by FlowCAM imaging flow cytometer and microscopy.

Phylum	Morphological group classified by FlowCam	Species identified by microscopy
Cyanobacteria	*Microcystis spp.*	*Microcystis spp.*
	Aphanocapsa spp.	*Aphanocapsa* cf. *delicatissima* West & G.S.West 1912
	Filamentous cyanobacteria	*Cuspidothrix issatschenkoi* (Usachev) P.Rajaniemi, Komárek, R.Willame, P. Hrouzek, K.Kastovská, L.Hoffmann & K.Sivonen 2005, *Pseudoanabaena* sp.
Chlorophyta	*Micractinium* spp.	*Micractinium pusillum* Fresenius 1858

	Scenedesmus spp.	*Tetradesmus lagerheimii* M.J.Wynne & Guiry 2016, *Desmodesmus bicellularis* (Chodat) S.S.An, T.Friedl & E.Hegewald 1999,<br>*Desmodesmus communis* (E.Hegewald) E.Hegewald 2000,<br>*Desmodesmus armatus* (Chodat) E.H.Hegewald 2000
	Pediastrum spp.	*Pediastrum duplex* Meyen 1829, *Pseudopediastrum boryanum* (Turpin) E.Hegewald 2005
	Unicellular green algae *Monoraphidium* spp.	*Monoraphidium griffithii* (Berkeley) Komárková-Legnerová 1969
	Sphaerocystis sp.	
Charophyta	*Closterium* spp.	
	Cosmarium sp.	
Cryptophyta	Cryptomonads	*Cryptomonas curvata* Ehrenberg 1832,<br>*Plagioselmis nannoplanctica* (Skuja) G.Novarino, I.A.N.Lucas & Morrall 1994
Miozoa	Dinoflagellates	
Bacillariophyta	Centric diatoms	*Stephanodiscus* sp., *Aulacoseira* sp.
	Pennate diatoms	*Nitzschia* sp.
Euglenozoa	Euglena	*Euglena* sp.

Table S4. SIMPER analysis results for different temperature tanks.

Groups	Average dissimilarity	Species	Group (AMB/A2) aver.abund	Group aver.abund (A2/ A2+50)	Av. diss	Diss/ SD	Contrib %	Cum.%
**AMB & A2**	77.1	Cyanobacteria	65	34.8	41.8	1.2	54.3	54.3
		Chlorophytes	25.1	25	30.3	1	39.3	93.5
**AMB & A2+50%**	92.9	Cyanobacteria	65	0.2	44.3	1.2	47.7	47.7
		Chlorophytes	25.1	4.9	37.6	1.1	40.5	88.2
**A2 & A2+50%**	91	Cyanobacteria	34.8	0.2	36.7	0.9	40.4	81
		Chlorophytes	24.9	4.9	36.9	1.1	40.6	40.6

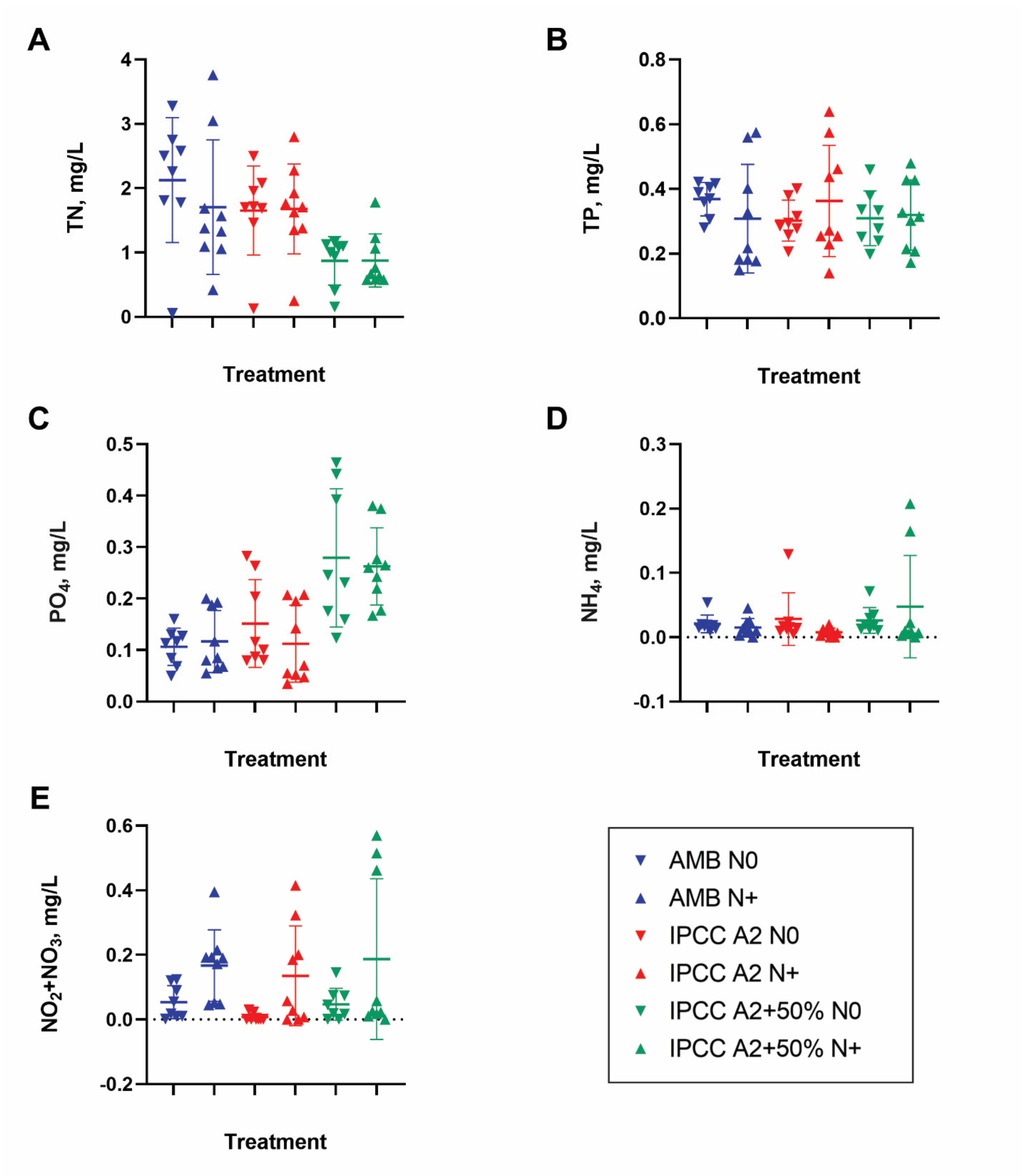

Figure S1. Concentrations of the main nutrients (TN, NH_4, NO_2+NO_3, TP, PO_4) in the tanks exposed to different temperature treatments (AMB, IPCC A2, IPCC A2+50%).

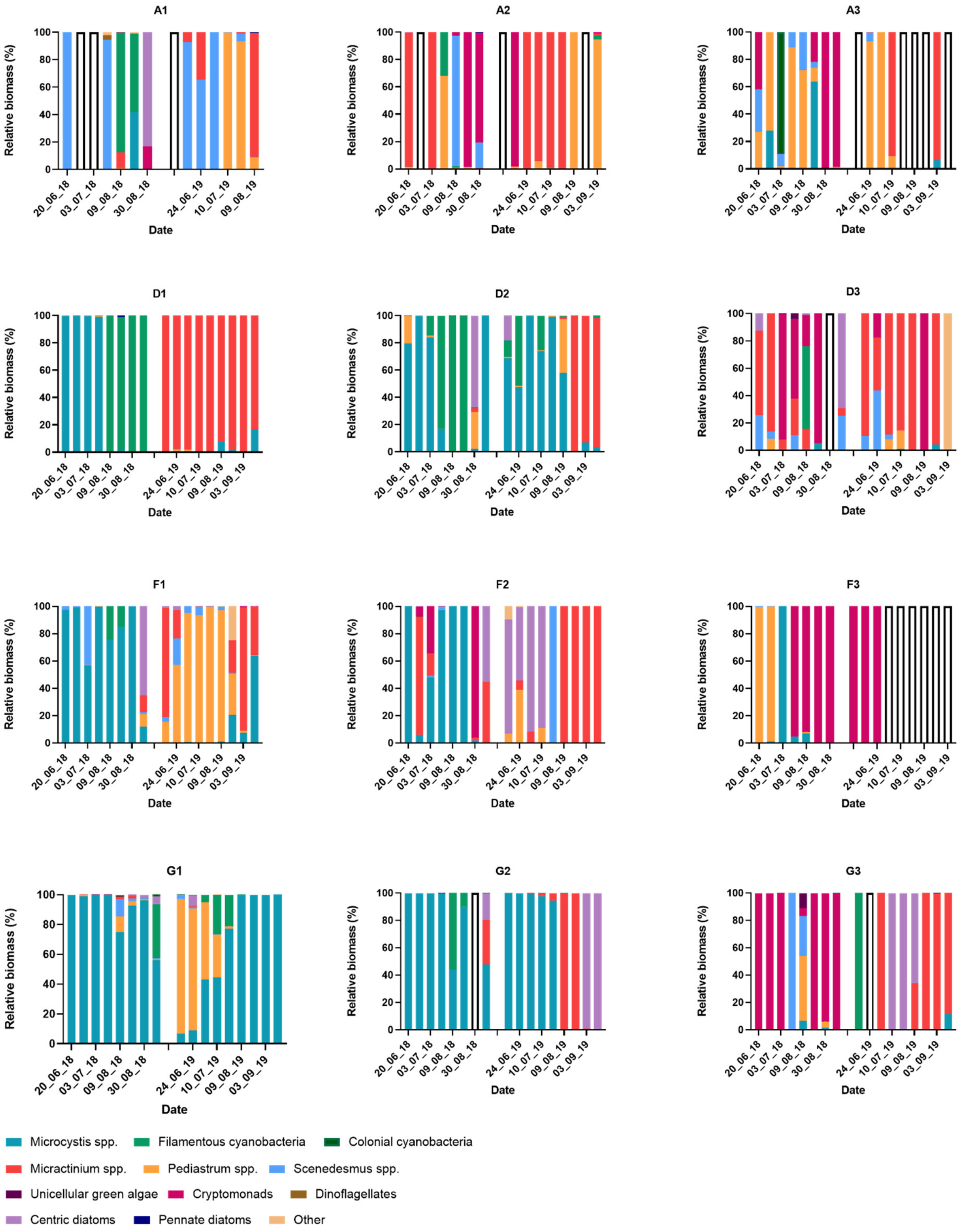

Figure S2. Relative contribution of the different phytoplankton groups to the total biomass in the individual tanks during the N0 and +N treatments.
